# Nonlinear model of cascade failure in weighted complex networks considering overloaded edges

**DOI:** 10.1038/s41598-020-69775-5

**Published:** 2020-08-10

**Authors:** Chao-Yang Chen, Yang Zhao, Jianxi Gao, Harry Eugene Stanley

**Affiliations:** 1 School of Information and Electrical Engineering, Hunan University of Science and Technology, Xiangtan, 411201 People’s Republic of China; 2 Center for Polymer Studies and Department of Physics, Boston University, Boston, MA 02215 USA; 3 Department of Computer Science, Rensselaer Polytechnic Institute, Troy, NY 12180 USA; 4 Network Science and Technology Center, Rensselaer Polytechnic Institute, Troy, NY 12180 USA

**Keywords:** Energy grids and networks, Complex networks, Complexity

## Abstract

Considering the elasticity of the real networks, the components in the network have a redundant capacity against the load, such as power grids, traffic networks and so on. Moreover, the interaction strength between nodes is often different. This paper proposes a novel nonlinear model of cascade failure in weighted complex networks considering overloaded edges to describe the redundant capacity for edges and capture the interaction strength of nodes. We fill this gap by studying a nonlinear weighted model of cascade failure with overloaded edges over synthetic and real weighted networks. The cascading failure model is constructed for the first time according to the overload coefficient, capacity parameter, weight coefficient, and distribution coefficient. Then through theoretical analysis, the conditions for stopping failure cascades are obtained, and the analysis shows the superiority of the constructed model. Finally, the cascading invulnerability is simulated in several typical network models and the US power grid. The results show that the model is a feasible and reasonable change of weight parameters, capacity coefficient, distribution coefficient, and overload coefficient can significantly improve the destructiveness of complex networks against cascade failure. Our methodology provides an efficacious reference for the control and prevention of cascading failures in many real networks.

## Introduction

With the advent of the network era, the network has become a part of people’s lives, many real-world networks can be abstracted as a complex network of nodes and edges^1^. For example, the transportation network^2, 3^, power gird^4–8^, information communication network^9, 10^, water supply network^11^ and so on. In such a network, even though an edge or a node fails, the entire network can be affected, often causing global collapse. A typical example is a massive blackout in the western United States in 2003^12^. Studying the cascading failures of complex networks can help us better understand the actual network. However, in actual dynamic networks, we often think that the components of the system have redundancy capabilities, and we can also understand that the system works under fluctuant load. So even if the loads exceed their capacity, it will only fail with a certain probability. Therefore, how to reduce this probability and distribute the excess load becomes an important issue in the study of network dynamics.

In rencent years, the robustness of networks against random and systematic component failures has attracted much attention. To understand the overall properties of the system, constructing the model is a good approach^13^. To build a cascade failure model, Motter and Lai (ML) proposed the model based on the relation between the load and the capacity, and they introduced a defense strategy to prevent cascading failures from spreading across the network^14^. When a random and systematic failures occurs in the network, a number of nodes are removed from the network and the load on it will be redistributed globally according to the shortest path strategy until the network reaches stability, Simulation results show when the load distribution exhibits heterogeneity, the deliberate attack will greatly reduce the robustness of the network, while the random attack is better, and the opposite results is the same. Inspired by the ML model, many studies have been devoted to the character and defensive measure of controlling cascaded failures^15–20^. For example, Wang and Kin (WK) proposed the WK model which defined the capacity as a monotone increasing function of the load, allocating additional capacity for high-load nodes. They found that if high-load nodes are protected, the network can be made more robust and cost at the same time^15^. In other words, the breakdown of a single node is sufficient to affect the entire system if the node is among the ones with largest load^16^. However, in the study of the actual network, it is found that the relation between the load and the capacity is not linear, and the edge with smaller load has more residual capacity^21^. We generally believe that the greater the robustness of the system, the higher the investment cost. So how to improve the robustness of the network as much as possible a limited cost is a question worth considering. The more common idea is to allocate a reasonable capacity for each node according to the needs of the system^22–24^, that is, the defined capacity is a nonlinear function with load as a variable. In this paper, we comprehensively consider the elasticity of the system, the relationship between the capacity and the load, and give a theoretical proof of the superiority of the nonlinear model. In addition, compared with the ML model, the simulation results show that the proposed model has the better robustness at the same cost. In the real world , the cascading failures often appear in a variety of forms, and evidence has shown that the impact on dependent networks is even greater.

With the dependent network theory model proposed by Buldyrev S.V in 2010, and its cascading failure process was analyzed, they also proposed that the strength of coupling reduces the robustness of the network^25^. On this basis, a large number of scholars have studied the dependency network from the perspective of cascading failure. At present, many models for the robustness study of the dependency network have been proposed^26–30^. Among them, Gao and Buldyrev S.V used the percolation theory to analyze the formation mechanism of the “network of networks”, showed that the proposed model is helpful for leading to a further analysis of real data on dependent networks and can be used to study the structure, function and robustness of dependent networks^26^. Considering the directionality of the real network, Liu and Stanley developed a theoretical framework based on the generation function and percolation theory and found that for different network models, the correlations between the in-degree and out-degree brings different results^28^. Based on a load-dependent cascading model, Zhong and Zhang simulated cascading failures when considering recovery strategies against, for different coupling strength and network topologies in interdependent networks^27^, In this paper, the overload status of the node is also considered, but only a value is set for it, and there is a lack of discussion about the redistribution of overload. As can be seen from the above literature, most researchers of them rarely consider the case of edges in the networks with redundancy capability when building the model of cascade failure, but in a real network, many connected edges often have some redundancy capabilities. Therefore, the modeling of cascading fault networks with redundant edges is worthy of further study. Therefor, the modeling of cascade failure networks with redundant edges is worthy of further study.

On the other side, comparing with modeling of weightless networks, it is more practical to describe real-world systems as the weighted networks in some cases. The weighted networks can positively affect some network’s characteristics. To discuss the relationship between weighting characteristics and robustness of the network, many weighting strategies about nodes or edges have been proposed, like a weighted strategy related to the degree^31^, betweeness^32^, product of endpoint degrees^33, 34^. It is also possible to weight its components according to the characteristics of the actual network. Huang and Zhang proposed a model of transit systems based on coupled map lattices to analyze the robustness of weighted networks, it is concluded that the weighted method of complex network based on passenger flow can significantly improve the tolerance of the system for random faults^8^. Ganesh made a weighted analysis of the Indian airport network and compared the differences between the formation mechanism of China’s transportation network and that of the global transportation network^35^. These weighting strategies tend to make the network more robust. Most of them used the local weight flow redistribution rule to study the weighted complex network. In this paper, we also use the local weight flow redistribution rule for our research, Through the simulation of four typical network models, we found that the robustness of weighted networks is related to the heterogeneity of networks. Under the condition of paying the same cost, the robustness index of BA scale-free network rose the fastest, and this conclusion reversely shows that the heterogeneity of weighted edges is negatively correlated with the robustness of the network^36^.

Because of the elasticity of the real networks, the components of the network have a redundant capacity against load. It will not fail immediately when the load exceeds its own capacity. Moreover, for real networks, the strength of action between components and nonlinear characteristics of the network inevitably affect the cascading failure process. Therefore, it is necessary to study network cascading failure modeling that is more in line with the actual network characteristics and analyze the impact of various characteristics on the network robustness. This paper proposes a new cascading failure nonlinear model of a complex network considering overloaded and weighted edges. To make the cascade failure model more close to the actual network, the overload coefficient is used to describe the redundancy capability of edges, and the weight coefficient is used to describe the action intensity between nodes, the failure probability is used to describe the uncertainty of failure, and the capacity parameter is used to describe the non-linear relationship of different proportions. The results of analysis and simulation show that the model is a feasible and reasonable change of weight parameters, capacity coefficient, distribution coefficient, and overload coefficient and can significantly improve the resistance to cascade damage of complex networks.

## Results

Topology plays an important role in network dynamics research, and the typical topology helps to better understand and control the effects of cascading failures. There are different characteristics and structures in real networks, so this paper generates different topological structures for different situations.

First, we considered generating a scale-free network which proposed by Barabasi and Albert^37^, the rules for generating BA networks are mainly growth and priority connectivity, it means that the scale of the network is expanding and the new nodes are more inclined to the nodes with a high connection degree. Scale-free networks are widely observed in real-world, including transportation networks, the internet, world wide web, highway network etc.

The degree distribution of Scale-free networks is heterogeneous. However, there are a lot of homogeneous degree sequence in real-world, so this paper considers two other network models, ER network model and WS small world model. The construction of WS model is random reconnection on a rule graph, while the construction of ER model starts from the isolated nodes, with a certain probability connection between any two nodes^38^. Then, this paper generates a small-scale network model for verification(IEEE118 power grid).

While studying the theoretical model, this paper also considers the US power grid^39^ and applied the cascade failure to it.

Network parameters are shown in Table 1.

**Table 1:** Topology data of different networks.

Parameter	ER random network	WS netork	BA scale-free network	IEEE power grid	US power grid
The number of node	500	500	500	118	4941
System generation parameter	$p=4/499$	$p=0.1$	$m_{0}=2$, $m=2$	$m=179$	$m=6594$
$<k>$	4.0	4.0	4.0	3.3	2.7
A network of similar structures	America’s high-speed networks, Small Internet’s network	Power grid, software networks	American airlines networks, package networks	$\backslash$	$\backslash$

In view of the different characteristics of the real-world networks, this paper conducts simulation analysis for the above five models respectively, and takes the average of 50 times of independent simulation. This paper studies the damage resistance of a network from the aspects of overload coefficient, distribution coefficient, weight coefficient and capacity parameter.

### The influence of the overload coefficient $\delta$ on the damage resistance of network

**Figure 1:**
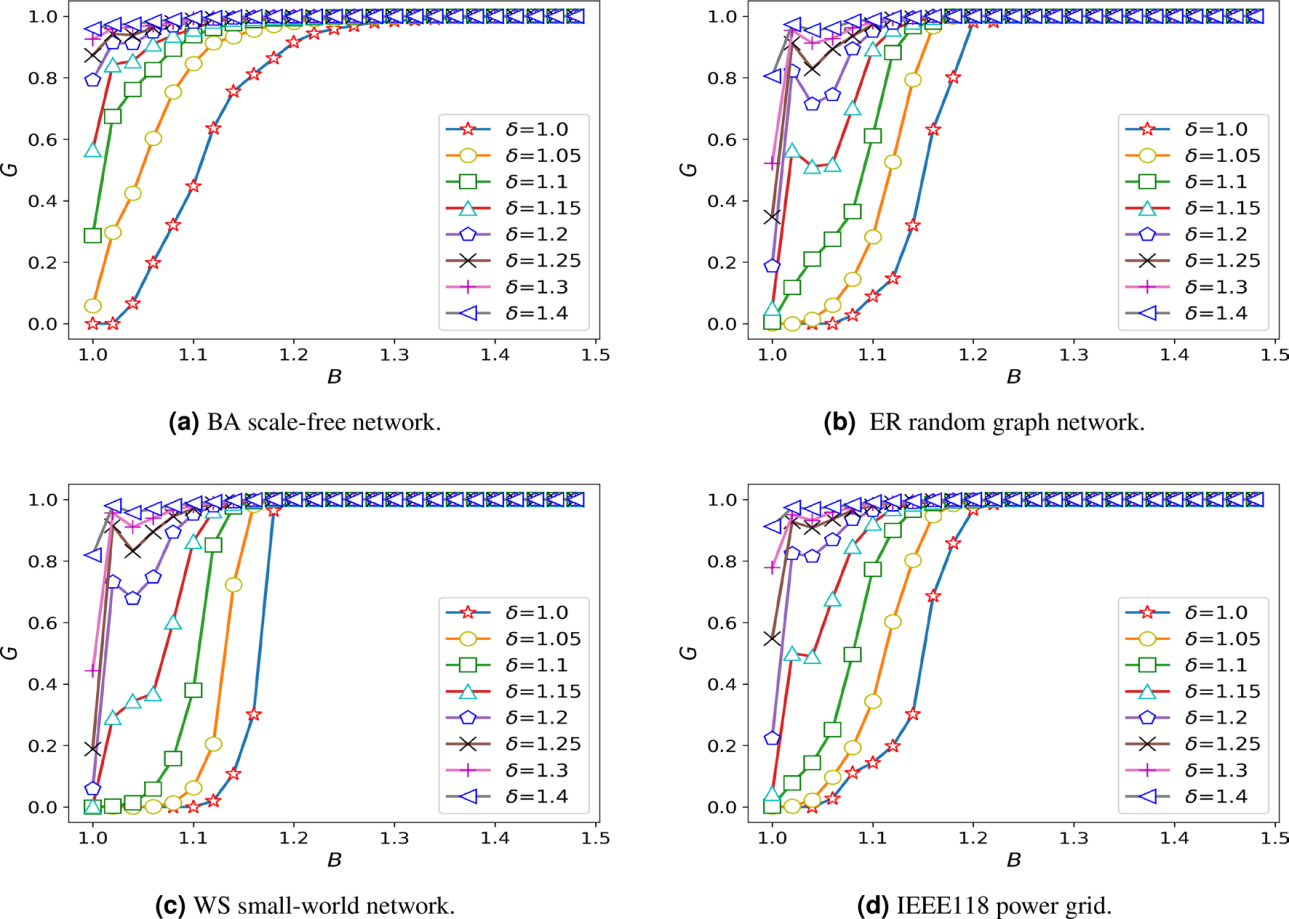
Diagram of damage resistance under different overload coefficients.

In order to explore the impact of overload coefficient on network damage resistance, the setting of other parameters should not have a great impact on the robustness. Through the study of other parameters, we find that the choice of distribution coefficient has little effect on the robustness of the system and when the $\beta$ is 0.2 and the value of $\alpha$ reaches 0.45, the robustness of BA scale-free network starts to change significantly. Meanwhile, we consider that the intensity of the action between the nodes should not be too large or too small, so it is assumed that the $\theta = 1$, $\alpha = 0.45$, $\omega = 1$. The overload coefficient is studied on different networks, and the simulation results are shown in Fig. 1.

**Figure 2:**
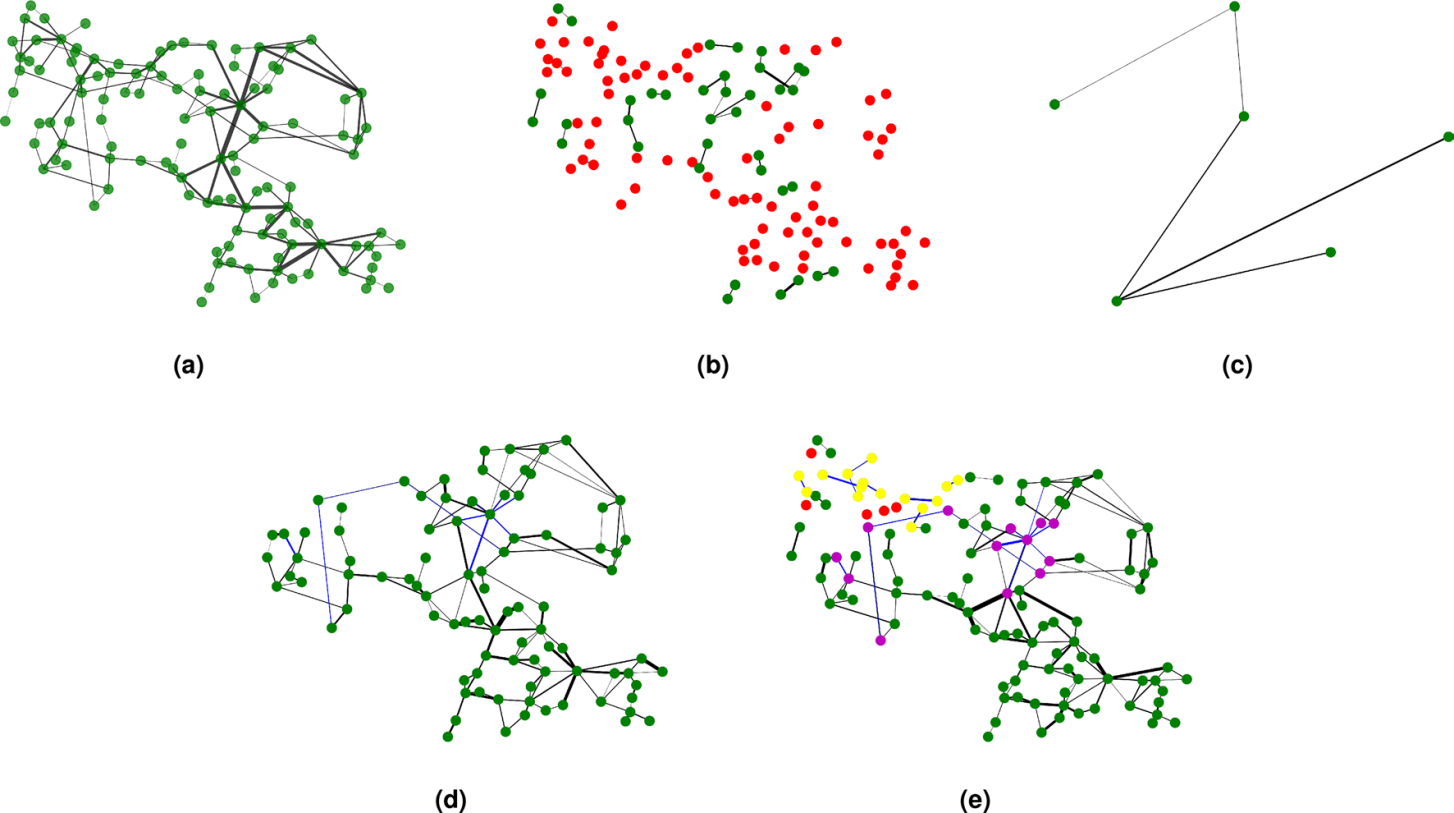
IEEE118 weighted power grid with different overload coefficients. **(a)** Original IEEE118 weighted power grid without failure: the green nodes represent the normal components of the grid, the thickness of the line represents the strength of the relationship between nodes and there is no fault in the grid. **(b)** The overload coefficient $\delta$ is not taken into account: attack one edge randomly to make it invalid. The red nodes represent the failed components of the grid. **(c)** Maximum connected subgraph of $\delta =1$: there are only six components that are still active in the grid. **(d)** The $\delta =1.2$: attack one edge randomly to make it invalid. the yellow and purple represent the overload nodes respectively, but the yellow nodes eventually fail in the grid because it is not in the maximum connected subgraph. **(e)** Maximum connected subgraph of $\delta =1.2$: at this time, most nodes in the grid can still work normally.

Through the analysis of Fig. 1, when the whole system does not pay a high cost, the system has the worst damage resistance when the overload state is not considered, and with the increase of overload coefficient in a small range can significantly improve the damage resistance of the system. However, with the improvement of the whole network’s damage resistance to the maximum, the increase of overload coefficient does not change the invulnerability of the network. Then, it can be seen from Fig. 1 that BA scale-free network is more sensitive to overload coefficient, and it can achieve stable state faster under the condition of increasing the same overload coefficient. As shown in the figure, BA scale-free network reaches the most robust state when $\delta = 1.3$, while WS small-world network, ER random graph network and IEEE118 power grid reach the strongest state when $\delta = 1.4$. From Fig. 1, we find that the network robustness and overload coefficient have the strongest relationship when *B* is 1.1, To describe the effect of overload coefficient on network robustness better, we assume that $B=1.1$, $\alpha =0.45$, $\omega =1$. Taking IEEE118 power grid as an example, analyzing the change of the maximum link of the network when attacking an edge at random in the case of not considering the overload coefficient and overload coefficient of 1.2 respectively, and the simulation results are shown in Fig. 2.

It can be seen from the figure that when a edge of the network is attacked randomly, there are only six nodes in the maximum connected graph when overload is not considered. With the increase of overload coefficient, the robustness of the network is greatly improved. The existence of overload makes the network continue to run well.

### The influence of the weighted coefficient $\theta$ on the damage resistance of network

**Figure 3:**
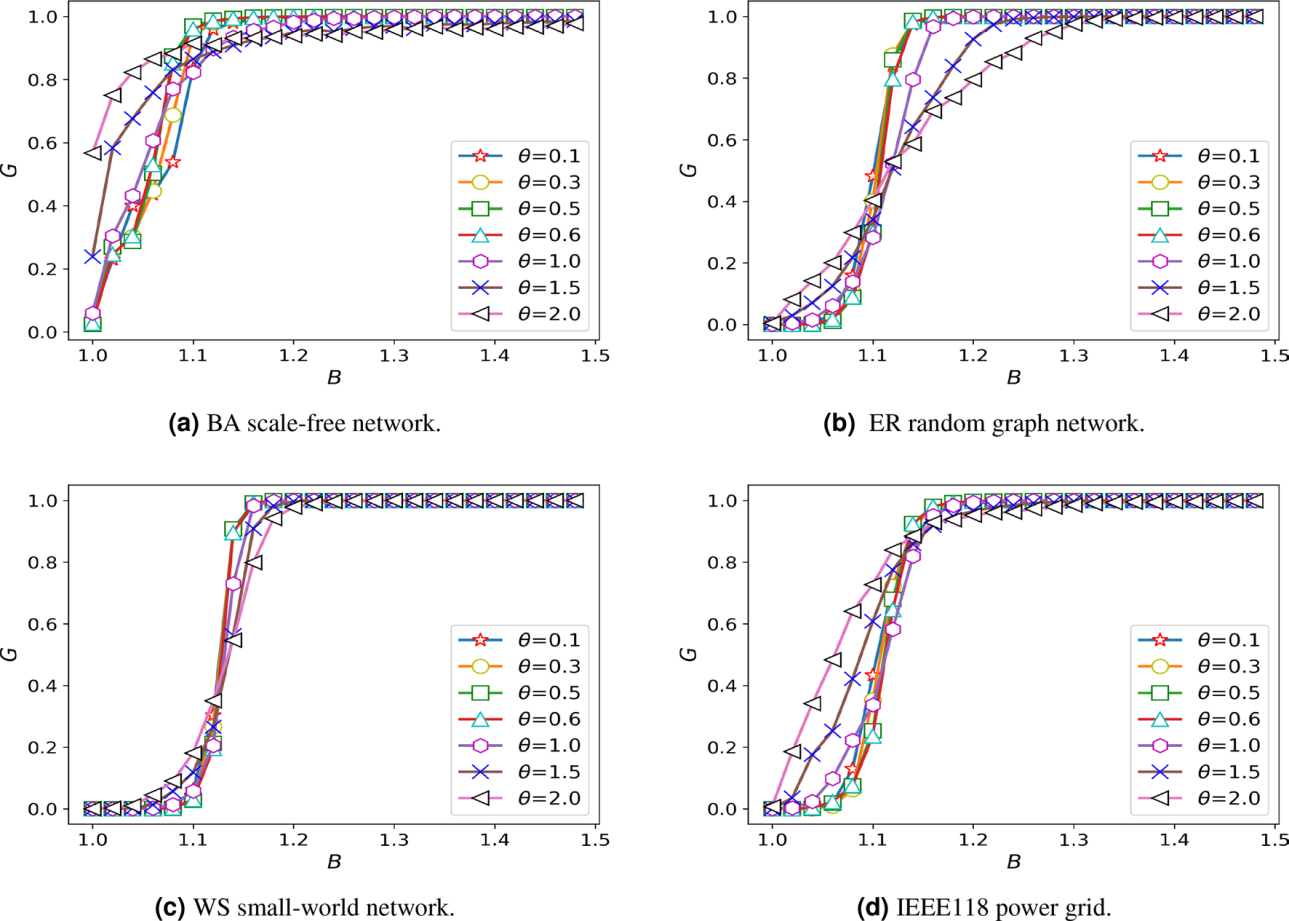
Diagram of damage resistance under different weighted coefficients.

In order to explore the impact of the weight coefficient on network damage resistance, the setting of other parameters should not have a great impact on the robustness. In the case of discussing the overload coefficient, we choose the minimum value with overload discussed in this paper and we assume that the $\delta =1.05$, $\alpha =0.45$, $\omega =1$. The weight coefficient is studied on different networks, and the simulation results are shown in Fig. 3

**Figure 4:**
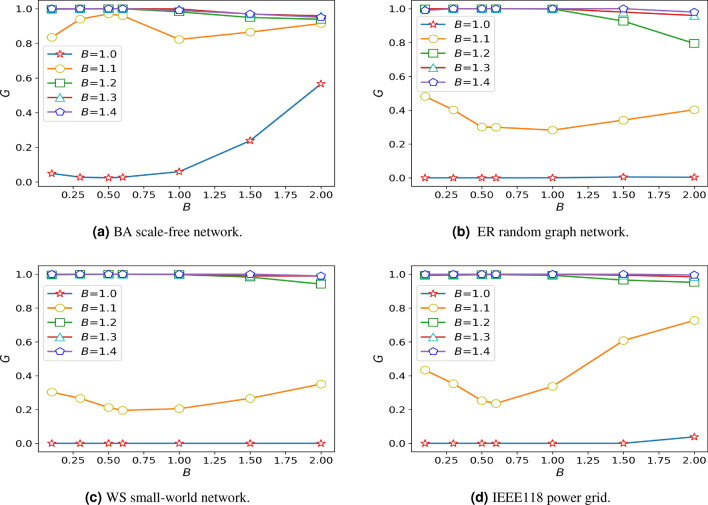
The resistance curve changes with the weighted coefficient under the different cost.

AS can be seen from the Fig. 3, the robustness of BA scale-free networks grows faster as costs increase. This phenomenon shows that the heterogeneity of weights is related to robustness from another perspective. The influence of weight parameters on different costs is different. Therefore, to reflect the impact of the weighted coefficient on a network, we will analyze the impact of the weighted coefficient on different network models at the same cost, and the results are shown in Fig. 4.

Due to the increased cost, the invulnerability of the system gradually strengthened. Firstly, when cascade failure does not occur in the network and the $\theta$ is greater than a turning point, the invulnerability of the system will decrease with the increase of the $\theta$. However, this point will be pushed back as costs rise. Among them, the decreasing trend of the ER random graph network is more obvious. When the cost is not sufficient to eliminate the small cascade failures, for BA scale-free network, when the $\theta$ is about 0.5, the system has the strongest damage resistance, and when the $\theta$ at around 1.0, the system has the worst damage resistance. When there is a large-scale cascading failure of the network, in ER random graph network and IEEE188 power grid, when the $\theta$ is less than 0.6, the invulnerability of system will decrease with the increase of the $\theta$, instead, the invulnerability of system will increase with the increase of the $\theta$, and the effect is obvious. The same rule is found in WS small world network, but the effect is not significant.

As can be seen from the Figure, the network dynamics behavior of IEEE188 power grid is more similar to that of ER random graph network and WS small-world network when the damage resistance of the system is similar.

### The influence of the distribution coefficient $\omega$ on the damage resistance of network

**Figure 5:**
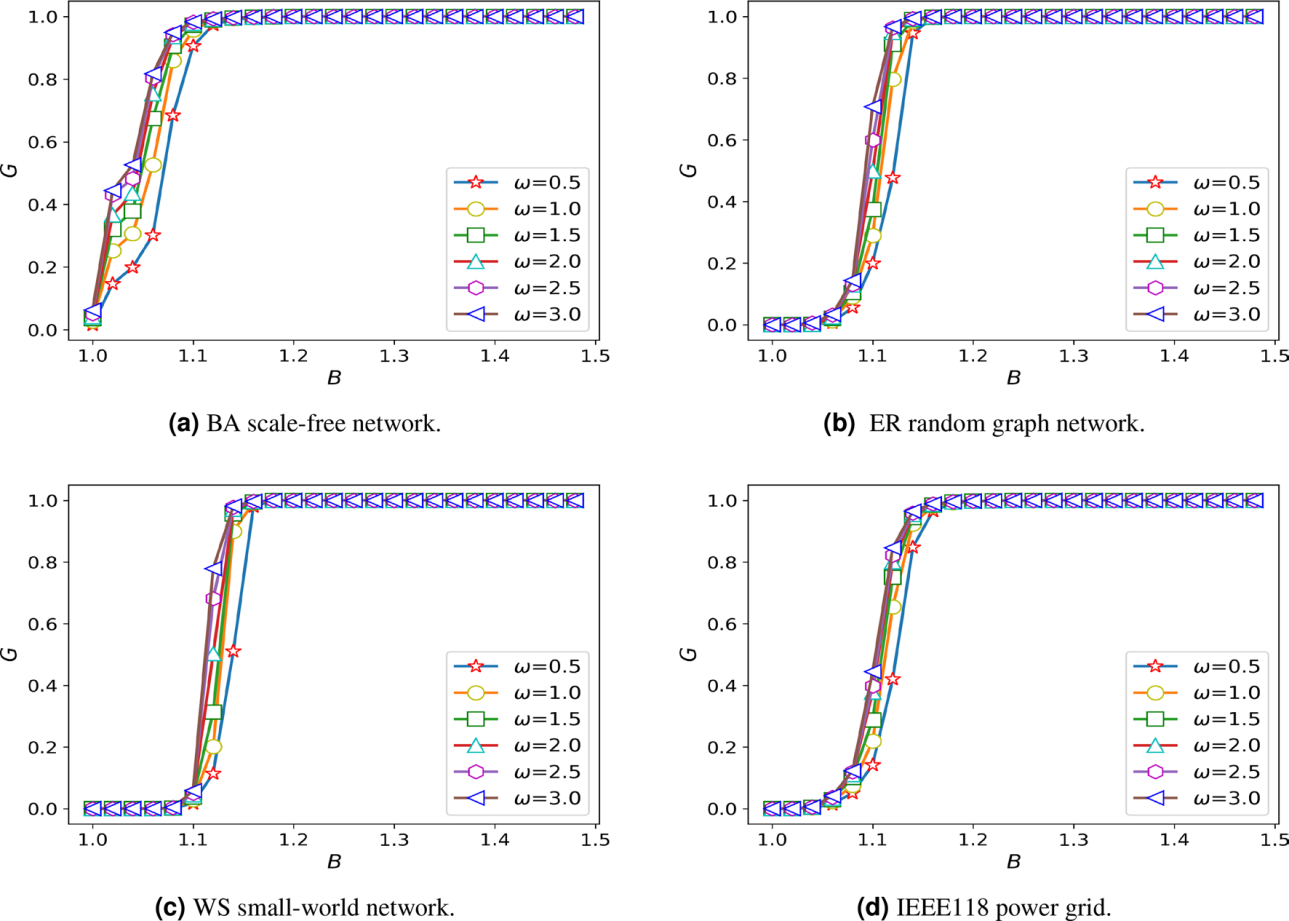
Diagram of damage resistance under different distribution coefficients.

Figure 4 shows that the robustness of the system is taken to an extreme value when $\theta =0.5$ or $\theta =0.6$, so taking these values can highlight the influence of the studied parameters on the robustness. To explore the impact of the distribution coefficient on network damage resistance, we assume that the $\delta =1.05$, $\alpha =0.45$, $\theta =0.5$. The distribution coefficient is studied on different networks, and the simulation results are shown in Fig. 5.

As shown in the pictures, the invulnerability of several networks will increase with the increase of the $\omega$, It is especially significant in BA scale-free network. As the system reaches its maximum resistance, the change of $\omega$ ill no longer causes the change of the damage resistance.

Within a certain range, the system becomes more invulnerability with the increase of the distribution coefficient in the research of the overall trend of several networks, which caused by the system have a higher failure probability even if the load exceeds the capacity a little when the distribution coefficient is small.

### The influence of capacity parameter $\alpha$ and $\beta$ on the damage resistance of network

**Figure 6:**
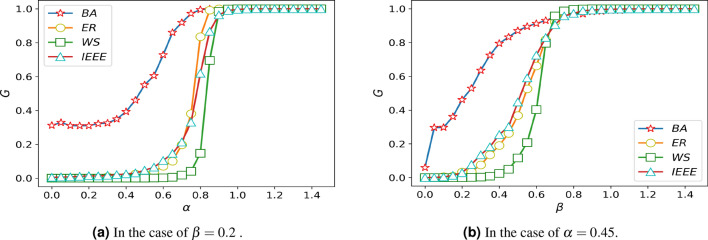
Diagram of damage resistance under different capacity parameters.

To explore the impact of capacity parameter on network damage resistance, parameter setting is consistent with the above analysis, so we assume that the $\delta =1.05$ , $\omega =1$, $\theta =0.6$. The capacity parameter is studied on different networks, and the simulation results are shown in Fig. 6.

As shown in the figures, the robustness of the system continues to strengthen with the increase of capacity parameter $\alpha$ in networks, there is a critical value $\alpha =1.0$ that the system has the strongest robustness. Similarly, the influence of $\beta$ on the damage resistance of network is similar to $\alpha$. Moreover, we can see that the BA scale-free network’s robustness begins to grow at a lower turning point, but it is growing more slowly than other networks.

When the average cost remains unchanged, the increase of capacity parameters $\alpha$ will lead to the decrease of capacity parameters $\beta$, which is negatively correlated with each other. The cost of the network will increases as $\alpha$ and $\beta$ increase. Therefore, it is not advisable to increase the capacity parameter blindly.

### Simulation based on actual network

Considering the cascading phenomenon of the actual network, we choose the US power grid as the research object for simulation analysis, and study the influence of overload coefficient and weight parameters on the cascading failure process. We find that compared with the nonlinear model without considering the overload (model 1) and the ML model (model 2) , the nonlinear model proposed in this paper that considers overload (model 3) is more robust at the same cost.

**Figure 7:**
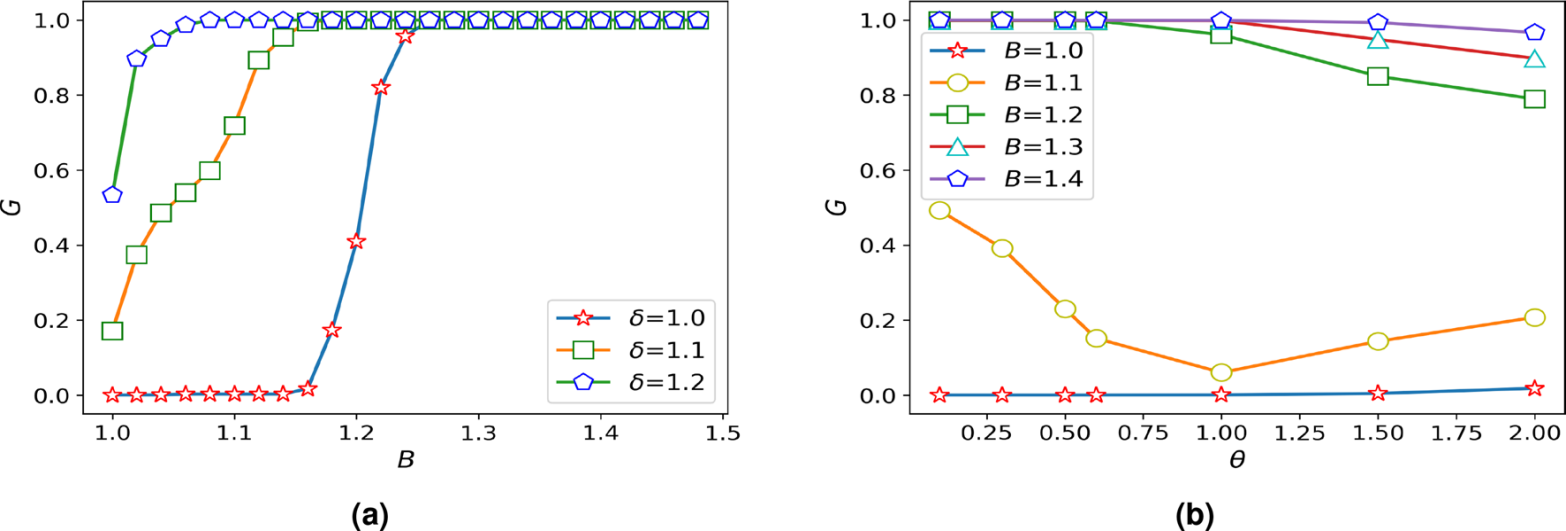
Diagram of damage resistance under different parameters in US power grid. **(a)** Diagram of damage resistance under different overload coefficients: we assume that the $\theta = 1$, $\alpha = 0.45$, $\omega = 1$. **(b)** The resistance curve changes with the weighted coefficient under the different cost: we assume that the $\delta =1.05$, $\alpha =0.45$, $\theta =0.5$.

The cascade failure simulation results of the model in the US power grid in this paper are shown in Fig. 7. It can be seen that with the increase of overload coefficient, the robustness of the system becomes better, but with the continuous increase of cost, the effect brought by the increase of overload coefficient will be weakened. The influence of the weight coefficient is similar to that of the appeal in IEEE118 network. when cascade failure does not occur in the network, when the $\theta$ is greater than a turning point, the invulnerability of the system will decrease with the increase of the $\theta$. However, this point will be pushed back as costs rise. When the network is in an unstable state, the robustness of the network has a process of first decreasing and then increasing as the weight coefficient increases, and the minimum value is obtained when $\theta =1$. Therefore, we can select characteristic model parameters according to the requirement of robustness.

**Figure 8:**
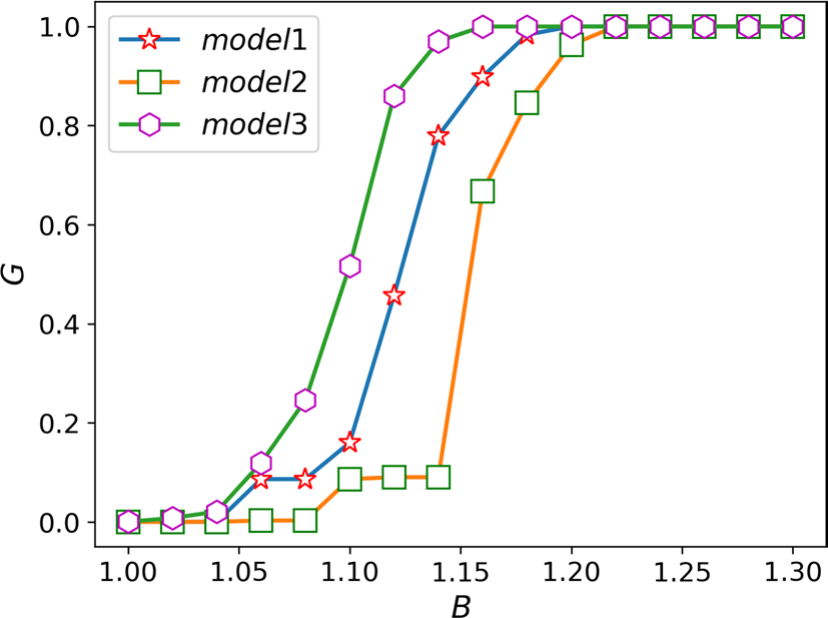
Cost and robustness comparison of the three models on the US power grid.

Figure 8 shows the relationship between investment costs of different models and network robustness. Compared with model 1 and model 2, the robustness of model 3 starts to increase earlier and reaches the maximum earlier, which can maximize the network’s resistance to cascading failures under the same robustness. This shows that the model we proposed is more suitable for practical networks and has practical guiding significance.

## Discussion

This paper discusses the overload mechanism of edges, considering the weighted characteristics of real networks and the nonlinear relationship between load and capacity, a cascade failure nonlinear model considering the overload and weighting characteristics of edges is proposed. And then, the following conclusions are obtained from four aspects: overload coefficient, weighted parameter, distribution coefficient, and capacity parameter.

1. If the overload coefficient is increased within a certain range, the invulnerability of the system will be increased, and in the BA scale-free network, the impact on the overload coefficient is more significant. However, when the overload coefficient is increased to a certain extent or the system cost is increased to a point where cascade failure does not occur, the increase of the overload coefficient will be meaningless.
2. When the distribution coefficient is less than 2.5, even if the side load exceeds a small part of the capacity, there is a high failure probability of failure. With the increase of the distribution coefficient, the invulnerability of the system gradually increases. However, when the distribution coefficient is increased to 2.5, continuing to increase the distribution coefficient will no longer have an impact on the failure resistance of the system.
3. When a large scale cascade failure occurs in the network at a low cost, the invulnerability of the network will increase with the increase of the weight parameters, but the effect is not obvious. With the increase of network cost, when the invulnerability of the network is about 50%, the damage resistance of a network decreases first and then increases with the increase of weight parameters. When cascade failure does not occur, after a certain weight parameter, the invulnerability of the network will decrease with the increase of weight parameters.
4. With the increase of the capacity coefficient, the robustness of the system is gradually strengthened. When a certain value is reached, it is no longer recommended to continue to increase the capacity parameter.

## Methods

### A cascading failure nonlinear model

**Figure 9:**
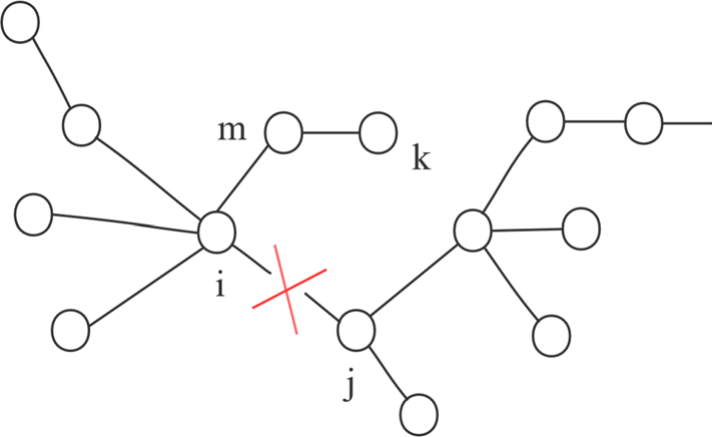
Schematic diagram of load distribution.

In urban traffic networks, some traffic roads have some elastic coefficients, when a load of a road is greater than its capacity, the road may not be completely blocked, but makes the system less efficient, as the load continues to increase, it becomes more and more vulnerable to failure. The model constructed in this paper is shown in Fig. 9.

As shown in Fig. 9, when the edge $e_{ij}$ fails, the load on it will be borne by other edges. In this paper, the weighted flow local redistribution principle^34^ is adopted, the weight of the node is related to its degree and the extra load assigned to each adjacent edge is positively correlated with its weight, so the load proportion that $e_{im}$ can share is:

1 $$\begin{aligned} \Delta L_{im}=\omega _{ij}\frac{\omega _{im}}{\sum _{a\in \eta }\omega _{ia}+\sum _{b\in \kappa }\omega _{jb}}, \end{aligned}$$

where $\omega _{ij}$ is the initial load of the $e_{ij}$, $\omega _{im}$ is the initial load of the $e_{im}$, $\eta$ is the set of all adjacent nodes of the node *i*, $\kappa$ is the set of all adjacent nodes of the node *j* .

If:

2 $$\begin{aligned} \Delta L_{im}+L_{im}> \delta C_{im}, \end{aligned}$$

the load on the edge is greater than the maximum capacity, the $e_{im}$ will fail and the load on it will be redistributed again until the cascading failure stops, where $\delta$ is the overload coefficient which is used to describe the capacity of edges to handle additional loads. the $C_{im}$ is the capacity of the $e_{im}$.

And if:

3 $$\begin{aligned} C_{im}< \Delta L_{im}+L_{im}< \delta C_{im}, \end{aligned}$$

the edge will be broken or overloaded based on a certain percentage, we can express it in the following formula:

4 $$\begin{aligned} \left\{ \begin{matrix} C_{im}< \Delta L_{im}+L_{im}< \delta C_{im}&{} rand> p_{im}\\ C_{im}< \Delta L_{im}+L_{im}< \delta C_{im}&{} rand\leqslant p_{im} \end{matrix}\right. \end{aligned}$$

where the rand is a random number between 0 and 1.

Each edge has a different capacity to handle the extra load. Sometimes, edges are more sensitive to overload in a small range, and the failure probability increases rapidly, however, there is also the case that the failure probability grows slowly. Therefore, we introduce a distribution coefficient of $\omega$ to characterize this feature. Namely, adjust the distribution of failure edge $p_{im}$ by the $\omega$, where the calculation of $p_{im}$ is as follow:

5 $$\begin{aligned} p_{im}=\left( \frac{L_{im}-C_{im}}{\delta C_{im}-C_{im}} \right) ^{\omega }. \end{aligned}$$

When an edge is overloaded, the edge does not fail, but the excess load of the edge needs to be evacuated quickly, so that the system runs normally. The specific expression as follows:

6 $$\begin{aligned} \Delta _{mk}=(L_{im}-C_{im})T_{mk}, \end{aligned}$$

where $\Delta _{mk}$ is the load distribution strategy of overloaded edges. This paper use the surplus load distribution^20^. the extra load assigned to each adjacent edge is positively correlated with its remaining load. Thus, we calculate it as follows:

7 $$\begin{aligned} T_{mk}=\frac{C_{mk}-L_{mk}}{\sum _{\alpha \in \Gamma _{i}}(C_{ia}-L_{ia})+\sum _{b\in \Gamma _{m}}(C_{mb}-L_{mb})}, \end{aligned}$$

where $\Gamma _{i}$ is all neighbor points of the node *i*, and the edges between this point and the node *i* are normal, $\Gamma _{m}$ is all neighbor points of node *m*, and the edges between this point and the node *m* are normal.

Then, in order to accurately describe the relationship between load and capacity in real-world, characterize its nonlinear distribution, this paper assumes the relationship between load and capacity is as follows:

8 $$\begin{aligned} C_{ij}=L_{ij}+\beta L_{ij}^{\alpha }, \end{aligned}$$

where $\alpha \geqslant 0$ and $\beta \ge 0$, when $\alpha = 1$, the model is degraded to ML^13^ model. the $L_{ij}$ satisfied:

9 $$\begin{aligned} L_{ij}=w_{ij}=(k_{i}k_{j})^{\theta }, \end{aligned}$$

where the $k_{i}$ and the $k_{j}$ is the degree of the vertex of the edge $e_{ij}$, the $\theta$ is the parameter for adjusting the load. By adjusting the load parameters, different network load distribution can be simulated.

In order to study the effect of a small initial attack on the cascading model and characterizes the state of edges overload, we cut an edge $e_{ij}$ and computed the number of normal edges and the edge of overload once the process of cascading failure is over. Then, we calculate the robustness value according to the following formula:

10 $$\begin{aligned} G=\frac{\sum _{h\in \Psi }S_{h}}{M(M-1)}, \end{aligned}$$

where *M* is the number of edges in the network, $\Psi$ refers to the set of edges that have not failed, when the edge state is normal, the $S_{h}=1$, and if the edge state is overload, the value of $S_{h}$ according to the following formula:

11 $$\begin{aligned} S_{h}=\frac{\delta C_{h}-L_{h}}{\delta C_{h}-C_{h}}. \end{aligned}$$

Increased robustness comes at cost. The cost of each edge is defined as the ratio of its capacity to its load, express as X, so the whole network’s cost defined as the average of X, express as B.

12 $$\begin{aligned} X_{ij}= & {} \frac{C_{ij}}{L_{ij}}, \end{aligned}$$

13 $$\begin{aligned} B= & {} \frac{1}{M}\sum X_{ij}, \end{aligned}$$

since it is impossible to improve the capacity of the network without limit in the actual network, the cost of the network should be controlled within a reasonable range and it makes no sense to increase the cost of the network when the network is robust.

### Theoretical analysis

To prevent cascade failure from proceeding further, the propagation of cascade failure is prevented in the first time. There are two cases:

1. When $\Delta L_{im}+L_{im}\leqslant C_{im}$, the cascade failure stopped.
2. When $C_{im}< \Delta L_{im}+L_{im}< \delta C_{im}$ and $rand> p_{im}$, the edge is overload which does not invalidate a new edge, the cascade failure stopped.

In case 1, substitute Eqs. (1), (8), (9) into equation, we can get:

14 $$\begin{aligned}&\frac{(k_{i}k_{j})^{\theta }}{\sum _{\alpha \in \eta }(k_{j}k_{\alpha })^{\theta }+\sum _{b\in \kappa }(k_{i}k_{b})^{\theta }}+1\leqslant 1+\beta (k_{i}k_{m})^{\theta (\alpha -1)}=X_{im}, \end{aligned}$$

15 $$(k_{i} k_{b} )^{\theta } = k_{i}^{\theta } \sum\limits_{{k{^{\prime}} = k_{{\min }} }}^{{k_{{\max }} }} {k_{i} } p(k{^{\prime}}|k_{i} )k{^{\prime}}^{\theta }.$$

where the $p(k'|k_{i})$ represents the conditional probability that vertex *i* of degree *k* is connected to vertex *i* of degree $k'$ and there is no degree-degree correlation when networks constructed through preferential attachment and Newman-Watts algorithms^34^, and many of the network degree correlations in reality are so small that they can be ignored. Moreover, most of the relevant researches of network science do not consider degree correlations. Therefore, Without losing generality, we ignore degree correlations in this paper, so we can get that:

16 $$\begin{aligned} p(k'|k_{i})=p(k'), \end{aligned}$$

Take it into Eq. (15)

17 $$\begin{aligned} \sum _{b\in \kappa }(k_{i}k_{b})^{\theta }=\frac{k_{i}^{\theta +1}<k^{\theta +1}>}{<k>}, \end{aligned}$$

and then take it into Eq. (13) and use inequality analysis to get:

18 $$\begin{aligned} 1+\frac{<k>}{2<k^{\theta +1}>}(k_{i}k_{j})^{\frac{\theta -1}{2}}\le 1+\beta (k_{i}k_{j})^{\theta (\alpha -1)}=X_{im}, \end{aligned}$$

following formula is made by the data proceeding and calculation based on the appeal equation:

19 $$\begin{aligned} 1+\frac{<k>^{\theta }}{2<k^{\theta +1}>}\le 1+\beta <k>^{2\theta (\alpha -1)}=B. \end{aligned}$$

Similarly, if the model is linear, we can get:

20 $$\begin{aligned} C_{ij}=(1+t)L_{ij}. \end{aligned}$$

By the same logic we can derive that:

21 $$\begin{aligned} 1+\frac{<k>^{\theta }}{2<k^{\theta +1}>}\leqslant 1+t=B, \end{aligned}$$

where the t in linear model is a constant value, which will lead to excessive extra capacity on the larger side of the load, and insufficient capacity on the smaller side, which will reduce the robustness of the system. So the nonlinear model can deal with the problem of uneven load distribution in real network more effectively.

In case 2, $C_{im}< \Delta L_{im}+L_{im}< \delta C_{im}$ and $rand> p_{im}$. Similarly, we can simplify this to the following:

22 $$\begin{aligned} B=1+\beta<k>^{2\theta (\alpha -1)}<1+\frac{<k>^{\theta }}{2<k^{\theta +1}>}<=\delta (1+\beta <k>^{2\theta (\alpha -1)})=\delta B. \end{aligned}$$

Similarly, if the model is linear, we can get that:

23 $$\begin{aligned} B=1+t<1+\frac{<k>^{\theta }}{2<k^{\theta +1}>}<=\delta (1+t)=\delta B. \end{aligned}$$

At this time, when the edge is overloaded, the capacity on the edge needs to be evacuated. The overload edge adopts the residual load distribution strategy, that is, the load weight borne by the $e_{mk}$ satisfies the following formula:

24 $$\begin{aligned} T_{mk}= & {} \frac{C_{mk}-L_{mk}}{\sum _{\alpha \in \Gamma _{j}(C_{ia}-L_{ia})}+\sum _{b\in \Gamma _{m}(C_{mb}-L_{mb})}}, \end{aligned}$$

25 $$\begin{aligned} \Delta _{mk}= & {} (L_{im}-C_{im})T_{mk}. \end{aligned}$$

If $\Delta _{mk}+L_{mk}\le C_{mk}$, the cascade failure stopped, and if $C_{im}< \Delta L_{im}+L_{im}< \delta C_{im}$ and $rand> p_{im}$, at this point, the edge is overload, and then the overload operation is repeated to determine whether the edge finally fails.
